# A Stable Backup Routing Protocol for Wireless Ad Hoc Networks

**DOI:** 10.3390/s20236743

**Published:** 2020-11-25

**Authors:** Fan Zhang, Gangqiang Yang

**Affiliations:** 1 School of Information Engineering, Shandong Management University, Jinan 250357, China; 14438120190051@sdmu.edu.cn; 2 School of Information Science and Engineering, Shandong University, Qingdao 226237, China

**Keywords:** mobile Ad hoc networks, backup routing, packet delivery, bit error rate, improving stability

## Abstract

High-speed mobility and heavy-load traffic in mobile Ad hoc networks (MANET) may result in frequent topology changes and packet loss. To guarantee packet delivery, a novel stable backup routing (SBR) scheme is put forward in this paper, which consists of the establishment of backup routes and route maintenance. In SBR, backup routes are set up by overhearing MAC signals, and the bit error rate is considered in path selection for improving stability. To repair broken links effectively and reasonably, qualified backup routes are classified into three categories with different priorities, based on which the relevant nodes decide how to reconstruct the forwarding path. Extensive simulations demonstrate that our proposed method outperforms other comparable backup routing mechanisms in terms of packet delivery ratio, average delay and control overhead.

## 1. Introduction

**Figure 1:**
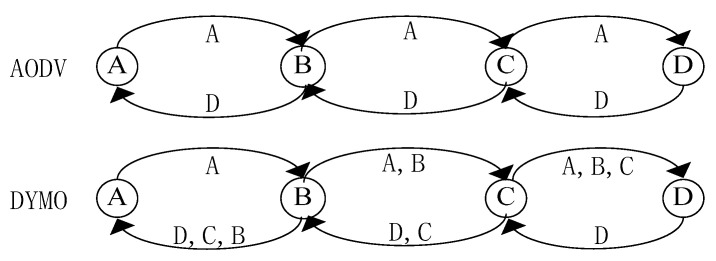
Route discovery in Ad hoc On-demand Distance Vector (AODV) protocol and Dynamic (Mobile Ad Hoc Network (MANET)) On-Demand (DYMO) routing.

A Mobile Ad Hoc Network (MANET) is formed by a collection of wireless mobile nodes without centralized administration [1]. Natures of self-organization, mobility and distributivity challenge the designing of the routing protocol for MANET. Most of the existing classical routing algorithms in Ad hoc networks are implemented based on smallest hop counts, such as the Ad hoc On-demand Distance Vector (AODV) protocol [2] and Dynamic MANET On-Demand (DYMO) routing [3]. Both AODV and DYMO have been published as Request For Comments (RFC) standards of Ad hoc network routing protocol by the IETF (Internet Engineering Task Force) working group. DYMO can be conceived as a successor to AODV. The most obvious feature distinguishing DYMO from AODV is the path accumulation function, which permits nodes listening to routing messages to acquire related information about paths to other nodes without initiating route discoveries themselves. The process is illustrated in Figure 1. In AODV, after the route request is replied, node A knows only the paths to B and D. However in DYMO, node A additionally obtains a route to node C. Clearly, the control overhead in DYMO is lower than that in AODV. However, DYMO may incur higher packet loss because of its relatively unreliable paths. By far, there have been some other types of standardized routing protocols in Ad hoc networks, such as table-driven routing and zone routing protocols. In contrast, on-demand routing protocol (such as AODV or DYMO) incurs lower overhead and has better adaptability to MANET environment with limited bandwidth. All of the routing strategies above are based on hop counts. That is, the shortest path from source to destination is preferentially selected as the data forwarding path in the process of route establishment.

Due to the node mobility, energy limitation and distribution characteristics of MANET, the shortest path may not be the best transmission route. Aiming to make the network routing protocol better adapt to the specific Ad hoc network and improve the network performance in different application scenarios, many researchers have analyzed and studied the Ad hoc network routing protocols from different angles in recent years, and put forward improvement methods [4,5,6,7,8,9,10,11,12,13,14,15,16]. Among them, some researchers set the research goal to reduce end-to-end delay, save energy and improve security [4,5,6,7,8,9]. The authors in [4] proposed a Trust-based Optimized Link State Routing mechanism (TUE-OLSR) to enhance the security of the mobile Ad hoc networks, which uses the performance metrics of a node to evaluate the trust value of the node. Riasudheen et al. [5] attempted to reduce the energy consumption by proposing an Energy-Efficient Cloud-Assisted Routing Mechanism (EECRM) for MANETs. Under EECRM, energy consumption is minimized by performing fast local route recovery among mobile nodes and peer nodes. The authors of [6] proposed a secure routing protocol based on AODV that successfully achieves both security and communication efficiency. In the proposed method, each intermediate node holds a packet received from a specific node in the past, and appends the held packet to the Route Request (RREQ) of another node and generates its own signed Route Reply (RREP). In [7], a novel routing protocol is proposed to construct reliable and energy-aware routes considering energy consumption of nodes in which the remaining energy is used as an important parameter for selection of a node in a route. The authors of [8] analyzed the transmission latency and signaling overhead in opportunistic routing. Shelke et al. [9] introduced a priority-based congestion control mechanism in AODV protocol, which reduced delay, improved delivery rate and throughput. In addition, the flooding mechanism used in the routing discovery process of on-demand routing protocols (such as AODV) may cause broadcast storm problems. In order to alleviate the broadcast storm, some researchers put forward improved broadcast schemes [10,11,12]. For cognitive packet networks, Gelenbe et al. [13,14,15,16] studied packet-based path forwarding strategies to meet specific Quality of Service (QoS) requirements and provided valuable experimental data, which motivated work on autonomic communications [17]. It is worth noting that the exciting research results above focused on the details of routing the algorithm itself, including routing selection and route maintenance. This paper focuses on improving network performance from another perspective, that is to use alternate paths to enhance stability, which differentiates our work from these research results.

**Figure 2:**
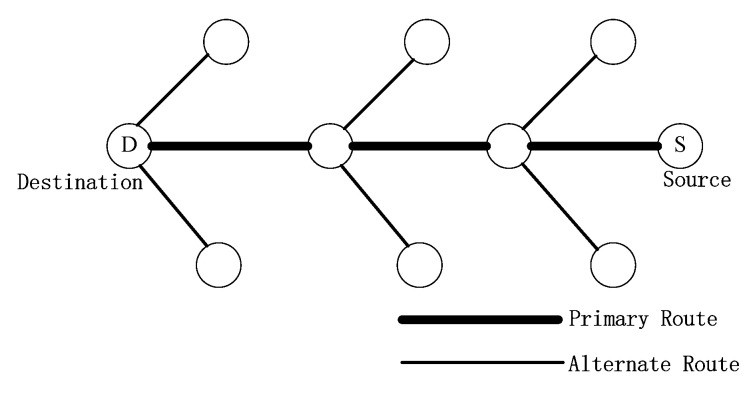
A fish bone structure formed in AODV-backup routing (BR).

In order to reduce the extra overhead and delay caused by frequent link breaking in MANET, designing more a stable routing protocol to transmit data with higher delivery is necessary. Research indicates that backup routing is an effective way to improve stability of networks. That is, if the primary path becomes invalid, data will be retransmitted through other backup routes, which definitely reduces the frequency of route rebuilding in comparison to single-path mechanisms [18]. In [19,20,21,22,23,24], these backup routes are established during route discovery process. E.g., AODV with backup routing (AODV-BR) [19] constructed an alternate path through overhearing RREP packets and a fish bone structure is formed jointly by the primary and alternate routes, which is illustrated in Figure 2. Correspondingly, backup routes in [21] are found by the destination node when the RREQ packets are received. It could be observed that such an approach usually finds all alternate routes within the scope of network topology. However, as nodes move faster, the backup paths generated only in route discovery process may be out of date and become unavailable, which may influence the reliability of data transmission.

To be adaptive to topology variations, alternative routes can be created through overhearing datagrams [25,26,27,28,29]. The authors in [25] proposed an AODV adaptive backup routing named as AODV-ABR without sending many extra control messages. In [26], a novel fast route recovery scheme was presented by overhearing both data and acknowledgements. Listening to data packets to collect the required information may lead to a huge amount of energy consumption because of the data length [30], which is the main shortcoming of such a method. Another strategy for building backup routings is implemented by overhearing MAC signals during the data delivery phase [30]. In [30], a source routing with local recovery (SLR) was presented. Under SLR, an intermediate node listens to request-to-send (RTS), clear-to-send (CTS) and acknowledgement (ACK) messages to update its MAC cache which is established for recording bypass routings. Similar to the schemes of overhearing datagrams, the approach that utilizes MAC signals to set up backup routes may not always discover all the existing candidates. Nevertheless, it has better adaptability to the mobility of nodes. It should be further noted that snooping of MAC headers does not incur any additional energy overhead since a node must try to overhear MAC frames to affirm if there is a data packet being transmitted to it in any case, which is the inherent mechanism of IEEE 802.11 [31]. However, unfortunately, all the existing research results on building alternate paths have not considered the stability of backup routes. In fact, alternate paths should not be selected carelessly because unstable links may cause the rebuilt route to be broken again.

In view of the discussion above, this paper aims to improve the stability of alternate paths. In this direction, a stable backup routing (SBR) scheme is presented. SBR constructs backup paths through overhearing signal exchanges at the MAC layer, which is like that in [30]. Different from other existing backup routing protocols, link stability is considered in our scheme, which depends on the bit error rate. In detail, we classify the qualified backup routes into three categories with have different priorities based on hop counts. According to both the priority and link stability, nodes choose suitable alternate paths when link breaking occurs. As described above, our proposed algorithm is perhaps most comparable to the SLR in [30], which also uses RTS/CTS signals to build backup routes. Compared with SLR, the SBR algorithm proposed in this paper uses the power information of the physical layer to set up more stable backup routes, which further improves the network performance. Simulation results verify our analysis.

The rest of article is organized as follows. Section II gives the algorithm and realization process of SBR. Simulation and analysis are introduced in Section III. Finally, we conclude in Section IV.

## 2. Stable Backup Routing Scheme (SBR)

### 2.1. System Model

**Figure 3:**
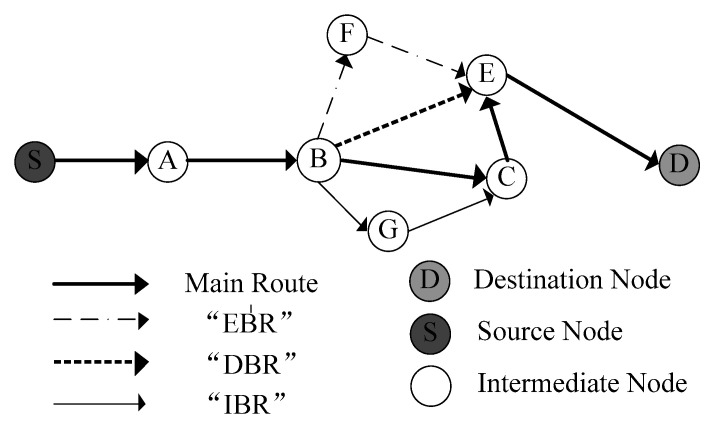
Three types of backup routes.

In the SBR, backup routes are classified into three categories based on hop counts or path length. As shown in Figure 3, there are three backup paths in the topology. The hop counts of the backup route through B–E decrease by one compared with that of main route, and such a path is called a decreased backup route (DBR). Similarly, because of the equal hop counts to that of the main route, the backup route through B–F–E is called an equal backup route (EBR). With regard to the backup route through B–G–C, whose length is larger than that of main route by one, it is named the increased backup route (IBR).

From Figure 3, we can see that “IBR” has the longest route to destination whereas “DBR” has the shortest path. Thus, one can select a different type of backup route to deliver packets according to practical application requirements. For instance, if real-time traffic (music or video streams) that require Quality of Service (QoS) support is being transmitted in Ad hoc networks, “DBR” or “EBR” should be chosen to meet the low delay requirement. For the purpose of shortening transmission delay, in SBR, “DBR” has the highest priority due to its smallest hop counts and the priority of “IBR” is the lowest.

To estimate link stability, the bit error rate (BER) is introduced which depends on the Signal to Noise Ratio (SNR) obtained from physical layer [32]. In this paper, the wireless Additive White Gaussian Noise (AWGN) channel and the Binary Phase Shift Keying (BPSK) digital modulation are used. Then the BER can be given by [33]

(1) $$\mathrm{BER}=\frac{1}{2}erfc\left(\sqrt{\frac{P_{r}\cdot B}{P_{N}\cdot T_{b}}}\right)$$

where P_r_ denotes the received power, B is the channel bandwidth, P_N_ represents the noise power, T_b_ is the transmission bit rate and *erfc* is the complementary error function.

Denote the link between node *i* and *j* by (*i*, *j*), the successful rate on link (*i*, *j*), named *p*^(*i*, *j*)^ is defined as:

(2) $$p^{\left(i,j\right)}={\left(1-BER^{\left(i,j\right)}\right)}^{l}\;,$$

where *l* is packet length and BER^(*i*, *j*)^ is the bit error rate of (*i*, *j*) calculated by (1). It is assumed that the channel is symmetric in this paper, i.e., *p*^(*i*, *j*)^ = *p*^(*j*, *i*)^. As we know, data transmissions in adjacent links interfere with each other, which can be avoided by the MAC layer’s RTS/CTS mechanism. The success rate *p*^(*i*, *j*)^ here refers to the probability that a packet is received correctly by node *j* after being sent by node *i*. Hence, the path successful rate is then the product of all passing links’ successful rates. Essentially, path successful rate defined here is a metric for evaluating the path quality, which indicates the probability for a certain packet to pass through a piece of route successfully. Note that, in addition to AWGN and BPSK, our proposed SBR can also be applied to other noise models and modulation methods without losing generality.

### 2.2. Backup Route Construction

Similarly to [30], backup routes in our SBR are established and updated by overhearing MAC signals during data transmission process. Different from the SLR algorithm proposed in reference [30], the SBR protocol selects backup routes that are more stable than the main route in terms of the path successful rate in order to guarantee the stability. 

**Figure 4:**
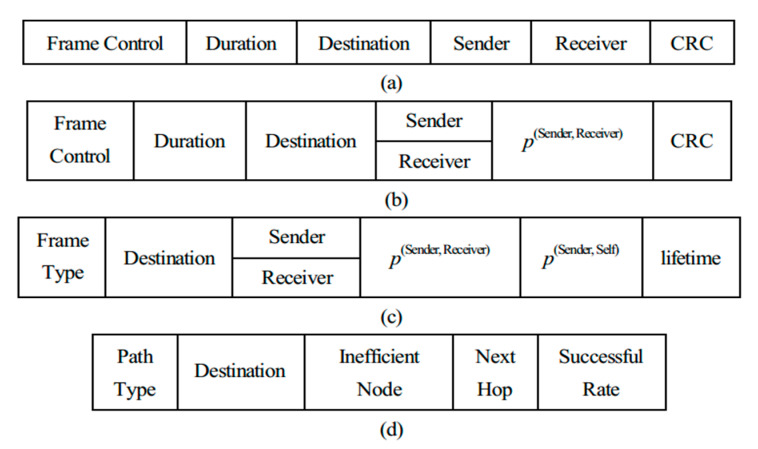
Structures of (**a**) request-to-send (RTS), (**b**) clear-to-send (CTS), (**c**) MAC cache, (**d**) backup route table.

For establishing backup routes, both the MAC cache and backup route table need to be constructed for each node in MANET, and the structures of RTS and CTS are also modified, which are described in Figure 4. As we can see from Figure 4a,b, both RTS and CTS messages carry the addresses of sender, receiver and destination. The difference is that *p*^(Sender, Receiver)^ calculated by (2) is appended to CTS, which is used to represent the stability of the main link between sender and receiver. The other fields such as frame control, duration and CRC are set in accordance with the standard IEEE 802.11 RTS/CTS formats. Note that the modified RTS and CTS include 26 and 28 bytes, respectively, whereas the lengths of legacy RTS and CTS are 20 and 14 bytes. The MAC cache in Figure 4c contains a series of entries, which records all the information obtained by overhearing RTS/CTS exchanges that are not for the node itself. Specifically, the item “Frame Type” is set as “RTS” or “CTS” according to the type of the MAC frame received. If RTS is overheard, *p*^(Sender, Self)^ is then calculated by (2) and *p*^(Sender, Receiver)^ should be “null” in the corresponding entry. On the other hand, if CTS is intercepted, *p*^(Sender, Self)^ is computed by (2) likewise, and *p*^(Sender, Receiver)^ is achieved or copied from the value in CTS. The existence of MAC cache provides evidence for setting up and updating backup route table. In the backup route table given by Figure 4d, “Path Type” means “IEB”, “EBR” or “DBR” and “inefficient node” is the possibly unreachable node due to link breaking. “Successful rate” stands for the stability of the alternate path. All of the entries in the MAC cache have a maximum survival lifetime to prevent being out of date. According to SBR, the qualified RTS or CTS just received overwrites the corresponding entry that already exists in MAC cache and the lifetime of the entry will be reset following such updating. If the renewed RTS/CTS cannot be received within the duration of the lifetime, it will be considered unreachable and the related entry in MAC cache will be deleted.

**Figure 5:**
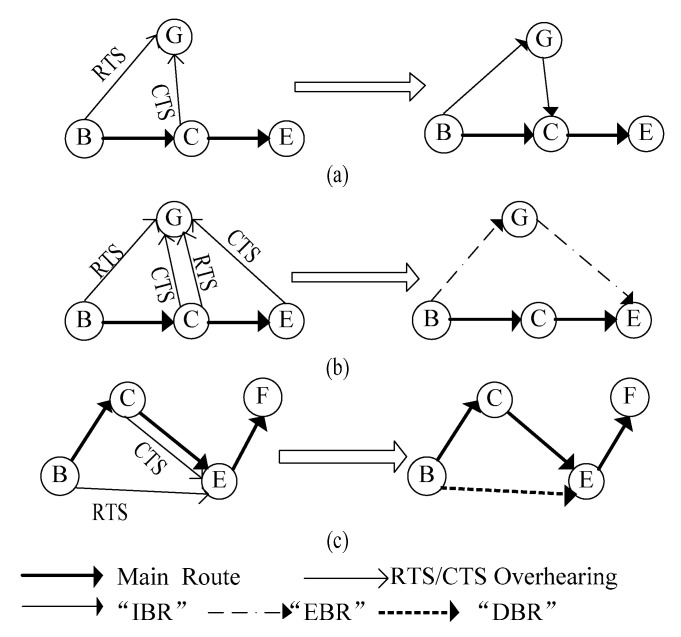
Establishment of (**a**) IBR, (**b**) EBR and (**c**) DBR.

We next explain how to obtain alternate routes based on the MAC cache. Establishment process of our three types of backup routes, i.e., IBR, EBR and DBR are illustrated in Figure 5, assuming that there is a path segment B–C–E–F on the main route to a specific destination and a node G that is located outside the main route. To facilitate the understanding without losing generality, we only set up alternate paths for link B–C in this example. For other links, execute them in the same fashion.

#### 2.2.1. IBR

In Figure 5a, node G traverses its MAC cache, if it preserves the information of RTS and responding CTS transmitted by B and C, respectively, and *p*^(B, G)^∙*p*^(C, G)^ > *p*^(B, C)^ is satisfied simultaneously, the “IBR” route entry will be appended into the backup route table of node G with “inefficient node” and “next hop” set as node C, “successful rate” as *p*^(B, G)^ ∙ *p*^(C, G)^. 

#### 2.2.2. EBR

As shown in Figure 5b, in addition to RTS/CTS information of link B–C, if G also cached RTS/CTS from the downstream link C–E, and with *p*^(B, C)^∙*p*^(C, E)^ < *p*^(B, G)^∙*p*^(G, E)^, then an alternate path “EBR” will be added to the backup route table of node G. At the moment, “inefficient node” is set as node C, “next hop” is E and the value of “successful rate” equals *p*^(B, G)^∙*p*^(G, E)^. 

#### 2.2.3. DBR

Different from IBE and EBR, DBR is constructed by the downstream node on the main route. In Figure 5c, node E has the main path to the destination itself. Through querying the MAC cache, if E overheard RTS/CTS exchange from upstream link B–C with *p*^(B, C)^∙*p*^(C, E)^ < *p*^(B, E)^, the “DBR” route entry will be appended into the backup route table of node E with “inefficient node” set as the address of C and “next hop” as F. The item “successful rate” is then assigned a value equivalent to *p*^(B, E)^.

Immediately after the entries of MAC cache for a certain destination change, the backup route table will be updated accordingly. For further ensuring timeliness, each node accesses its MAC cache periodically with a period P_a_ to update backup route table or delete unavailable alternate routes. It needs to be emphasized that a node at most maintains one backup route with highest priority for each user session according to the algorithm above. 

### 2.3. Backup Route Maintenance

In order to realize local link repairing and switch to backup routes automatically when link breaking occurs, two novel one hop control packets are created in SBR: backup route request (BRREQ) and backup route reply (BRREP). BRREQ contains destinations of the paths going through the broken link. Moreover, inefficient node address is appended into BRREQ to indicate which link needs to be repaired at present. As to BRREP, it is composed of path type, destination and successful rate derived from the backup route table.

Thus, the route maintenance algorithm in SBR is executed as follows: 

#### 2.3.1. Broadcasting BRREQ

When a node on the main route detects a broken link based on its MAC layer detection mechanism, it then broadcasts a BRREQ carrying the unreachable destinations and inefficient next-hop node caused by link breaking. At the same time, it waits a duration of T_th_ for replying. If no response is received after T_th_ expires, route rediscovery process will be performed according to the original routing protocol.

#### 2.3.2. Replying BRREP

After receiving BRREQ, each neighbor looks for available paths in its own backup route table by matching destinations and inefficient node included in BRREQ. Assume that a neighbor finds an alternate path to a certain destination, it would send a unicast BRREP to the sender of BRREQ to declare the existence of a backup route. The BRREP carries the necessary information of the corresponding backup route. Since the BRREQ/BRREP exchange is fast and occurs merely within the range of one-hop, the value of waiting time T_th_ only needs to be set to ensure timely receptions of all responding BRREPs. 

#### 2.3.3. Selecting Backup Route

It can be seen from the principle of SBR, the upstream node of the broken link may receive multiple BRREPs from different neighbors for a specific destination, which brings the selection problem of backup routes. In SBR, the node compares all the related BRREPs during the stipulated period T_th_ and gives DBR highest priority. Concretely, in order to ensure timeliness and stability simultaneously, path type is firstly compared and DBR, EBR, IBR has the priority from highest to lowest. Furthermore, if several usable alternate paths have the same type, the one whose successful rate is the largest wins.

After choosing backup route, the forwarding route table is then modified appropriately and data transmission will be switched from the main route to the selected backup route.

## 3. Simulation and Analysis

In this section, we perform a simulation experiment to compare the performance of the proposed SBR scheme with the previous work, AODV-SLR [30], using NS2-2.34. Through the analysis of Part I of the paper, the SBR algorithm proposed in this paper uses a similar way to AODV-SLR [30] to build backup routes. Meanwhile, algorithms that improve stability of the MANET involved in the literature [4,5,6,7,8,9,10,11,12,13,14,15,16] mainly focus on the routing protocol itself rather than backup routes. Therefore, we chose AODV-SLR algorithm as the comparison object of computer simulation. The AODV-SLR algorithm has the best comparability with the SBR presented in this paper. We integrate our SBR into AODV and DYMO, respectively, named as AODV-SBR and DYMO-SBR. In AODV-SBR, the main route discovery phase is fulfilled by AODV, whereas SBR takes responsibility for backup route construction and local link repairing. Similarly in DYMO-SBR, DYMO completes the main route building process using its path accumulation function. Actually, SBR can also cooperate with other QoS-guaranteed Ad hoc routing protocols, such as algorithms presented in [4,5,6,7,8,9,10,11,12,13,14,15,16], to further improve the network performance. The simulation parameters are shown in Table 1.

In our topology, each node randomly selects a position and moves toward that location with a random speed. Once it reaches that position, it becomes stationary for a predefined pause time. After that pause time, it selects another position and repeats the process. For each simulation, ten runs are carried out to obtain the average values of experiment results. The lifetime of all entries in the MAC cache and the accessing period P_a_ are both set as 10 s. The duration T_th_ that is used to wait for BRREPs is 50 ms. In the simulation, route performances are compared based on two scenarios: varying pause time from 0 to 300 s with CBR (Constant Bit Rate) = 4 packets/s and varying CBR data rate from 2 packets/s to 10 packets/s with pause time = 50 s. In practice, through the parameters set in Table 1 as well as the received/noise power from physical layer, the values of the BER and successful rates can be then calculated by (1) and (2).

We use three metrics to evaluate the algorithm performance, i.e., packet delivery ratio, average delay and control overhead. Specifically, packet delivery ratio is defined as the ratio of the number of delivered data packets to the total number of data packets generated by sources. Average delay is the sum of time taken by successful transmissions from sources to destinations divided by the total number of data packets delivered. Control overhead is defined as the number of all control packets required divided by the number of data packets successfully received.

**Figure 6:**
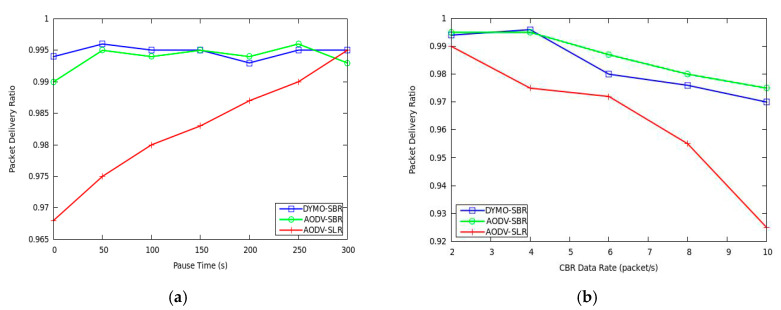
Packet delivery ratio versus (**a**) pause time and (**b**) CBR data rate.

Firstly, packet delivery ratio is compared by varying pause time and packet generation rate, respectively. From Figure 6a, it can be seen that AODV-SBR and DYMO-SBR outperform AODV-SLR especially when the topology changes frequently. If the topology is relatively stable, the frequency of link breaking is low and the performances of the three protocols are quite close. However, as pause time decreases, network topology changes more frequently, AODV-SLR does not guarantee packet delivery ratio any more. On the contrary, both AODV-SBR and DYMO-SBR select stable backup routes with lower packet error rate, and hence their performances improve a lot. Figure 6b shows that the packet delivery ratio of AODV-SLR is also lower than that of AODV-SBR and DYMO-SBR and the performance gap becomes more evident as the CBR data rate increases. The key reason is that when the network load becomes heavier, the probability of interference and collision suffered becomes higher and, hence, link breaking occurs more frequently. In SBR, the stability of backup routes is considered. Therefore, SBR guarantees transmission with better reliability and the packet loss decreases.

**Figure 7:**
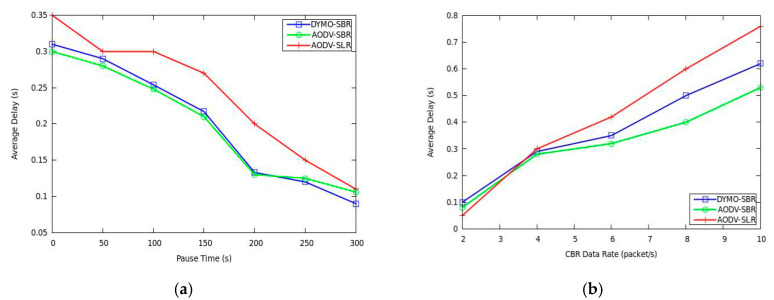
Average delay versus (**a**) pause time and (**b**) CBR data rate.

The results of average packet delay are demonstrated in Figure 7. As we can see from Figure 7a, the delay under our AODV-SBR and DYMO-SBR remains lower than that under AODV-SLR. The reason is that link condition is considered in SBR, which leads to better reliability. As we know, link breaking may force the routing protocol to initiate the path rebuilding process, which inevitably incurs extra transmission delay. Therefore, a more stable route means lower delay. Figure 7b reflects that AODV-SLR improves delay performance a little bit since it does not need to wait a period of T_th_ for selecting backup routes. However, as network load becomes heavier, the AODV-SBR and DYMO-SBR decrease transmission delay significantly. That is because the backup routes in SBR are more stable. Moreover, DBR is given highest priority to be selected to deliver packets, and thus the backup route in SBR may be shorter than that constructed by SLR. 

**Figure 8:**
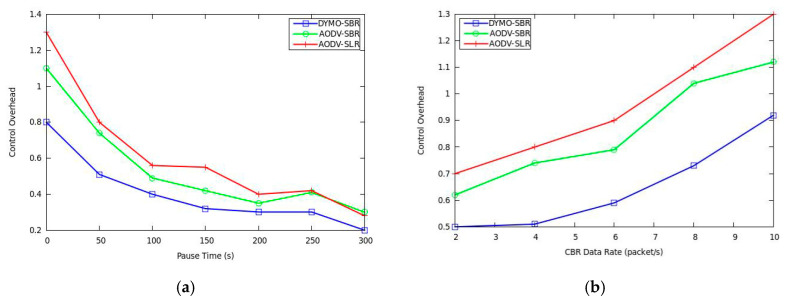
Control overhead versus (**a**) pause time and (**b**) CBR data rate.

Control overhead performance is shown in Figure 8, where we can observe that the proposed SBR has better performance with lower overhead. This is because SBR builds more stable backup routes. In fact, when link breaking happens, the processes of both backup route construction and original route rediscovery would bring additional routing cost. Therefore, improving route stability plays a key role in decreasing control overhead. In addition, from Figure 8, DYMO-SBR achieves the lowest control overhead, which benefits from its path accumulation scheme.

## 4. Conclusions

In this paper, a novel stable backup routing (SBR) scheme is developed, including procedures of backup route construction and route maintenance. Our backup routes are established by overhearing MAC signals. Due to the fact that the bit error rate of links is considered in building backup routes, the SBR decreases packet loss and improves the delivery ratio significantly. Moreover, in order to repair the broken link reasonably, all backup routes are classified into three categories with different priorities, which are used to ensure stability and shorten end to end delay simultaneously. Without losing generality, SBR can be integrated into the original Ad hoc routing protocols, such as AODV and DYMO. A mass of simulation experiments validate our theoretical analysis and demonstrate that SBR outperforms other comparable schemes.

In the era of 5G Internet of things, although 5G a cellular communication system with centralized architecture can ensure high bandwidth and low delay communication between the 5G terminal and base station, it cannot meet specific requirements of distributed network connection (such as Ad hoc, Wireless Sensor Networks, etc.). In the era of “ubiquitous networks and Internet of things”, the distributed networks based on its characteristics of no center and rapid deployment will provide a powerful supplement for a 5G cellular system. At the same time, when the base station cannot work normally due to war or natural disasters, the distributed mobile Ad hoc networks can also be used to ensure emergency communication. Therefore, how to optimize the existing wireless distributed network protocol architecture to meet the needs of low latency and high throughput under 5G background is worth further research.

In the next research work, more actual implementations and experiments will be conducted to obtain more valuable data and we will deeply study the MAC scheduling strategy based on low delay in distributed networks, combine with the routing protocol proposed in this paper, and use a cross-layer mechanism to further reduce transmission delay and improve network throughput.

## Figures and Tables

**Table 1:** Simulation parameters in the experiments.

Parameter	Value	Parameter	Value
Simulation time	300 s	Simulation terrain	1500 m × 300 m
Number of nodes	50	Mobility model	Random waypoint
Speed	0–20 m/s	Path loss model	Two-ray
Radio frequency	2.4 GHz	Bandwidth	2 MHz
MAC protocol	802.11	Transmission range	250 m
Entry lifetime	10 s	T_th_	50 ms
Pause time	0–300 s	CBR data rate	2–10 packets/s
CBR data sessions	0–20	Packet size	512 bytes
